# Supplementary material for: The CIN-TCP transcription factors regulate endocycle progression and pavement cell size by promoting cell wall pectin degradation
Source: Nat Commun. 2025 May 2;16:4108. doi: 10.1038/s41467-025-59336-7 (PMC12048579; doi:10.1038/s41467-025-59336-7)
Supplement: Supplementary file 1 — Supplementary Information [file 41467_2025_59336_MOESM1_ESM.pdf]

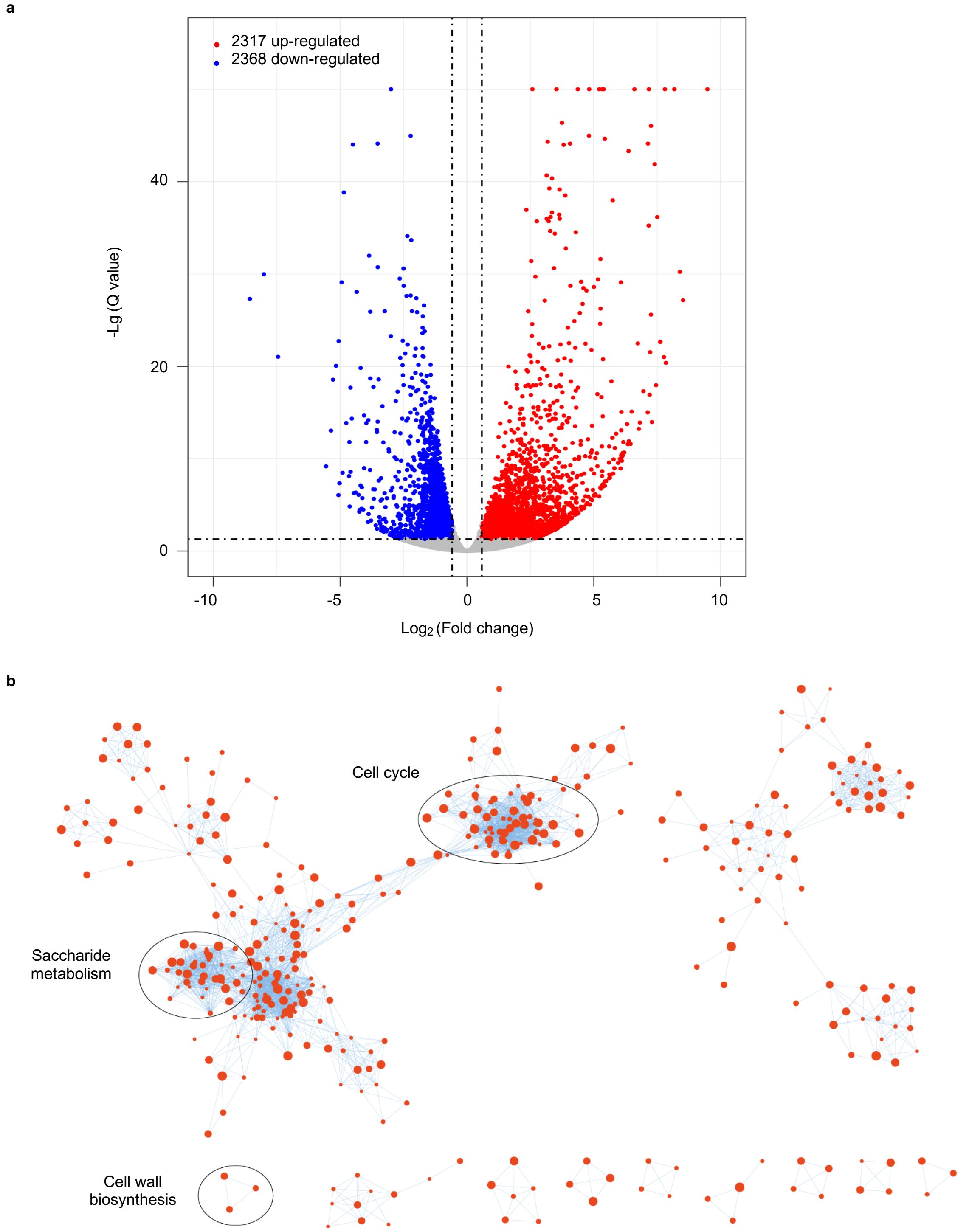

Supplementary Fig. 1 Analysis of differentially expressed genes regulated by CIN-TCPs. a Volcano plot showing the differentially expressed genes regulated by CIN-TCPs. The 2,317 up-regulated genes and 2,368 down-regulated genes in *tcpΔ7* versus the wild type are indicated in red and blue, respectively. **b** An association network of the enriched GO terms in the biological process category associated with the differentially expressed genes. Each red node represents an enriched GO term with FDR < 0.05. The sizes of the nodes reflect the degree of enrichment. The length of each blue line indicates the similarity between two GO terms.

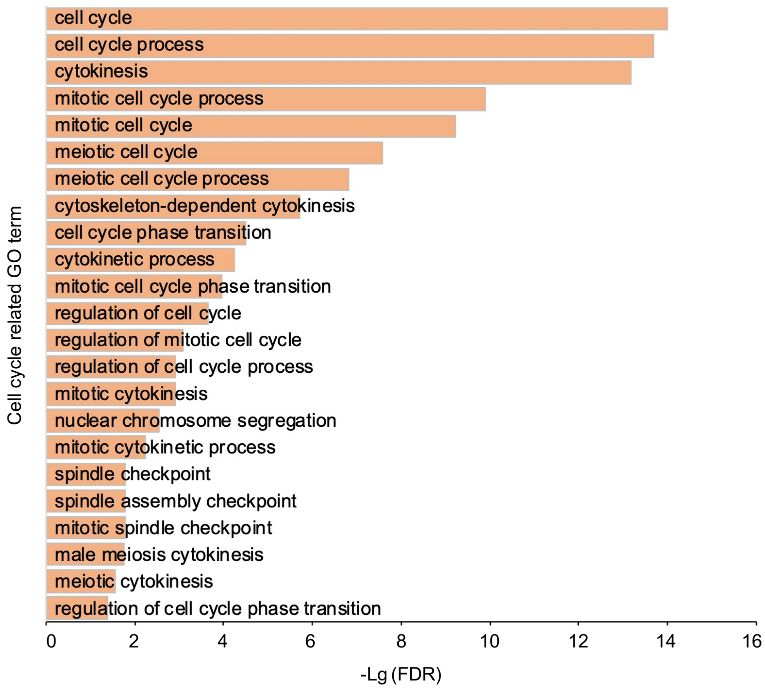

Supplementary Fig. 2 Enriched cell cycle related GO terms associated with genes regulated by CIN-TCPs. Enriched GO terms in the biological process category are shown. FDR was used to indicate significance of enrichment.

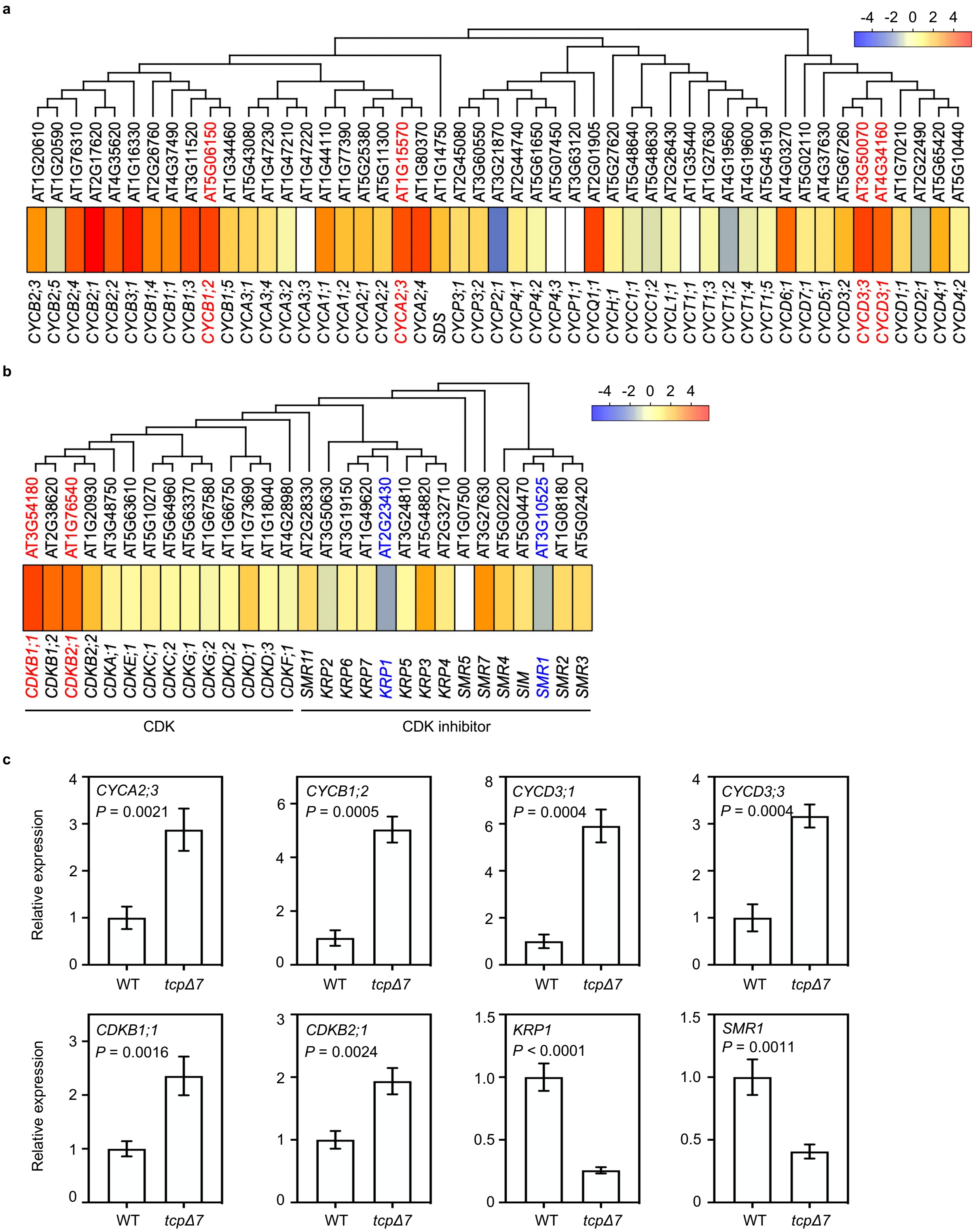

Supplementary Fig. 3 Expression profiles of cell cycle related genes. a Heatmap showing expression of the 49 *CYC* genes according to the RNA-seq data. Values are $\log_2$ fold change between *tcpΔ7* and the wild type. The genes further analyzed in **c** are indicated in red. **b** Heatmap of 14 *CDK* genes and 15 genes encoding CDK inhibitors. The genes further analyzed in **c** are indicated in red or blue. **c** Relative transcript abundance of eight selected genes in 7-day-old *tcpΔ7* and wild type cotyledons. *CYCA2;3*, *CYCB1;2*, *CYCD3;1*, *CYCD3;3*, *CDKB1;1* and *CDKB2;1* are positive regulators of the mitotic cell cycle. *KRP1* and *SMR1* are negative regulators of cell cycle. The expression level was determined by RT-qPCR analysis using *ACTIN7* as the internal control. Values are mean ± SD (*n* = 3 biological replicates). Statistical analysis was performed using a two-tailed unpaired Student's *t* test. Source data are provided as a Source Data file.

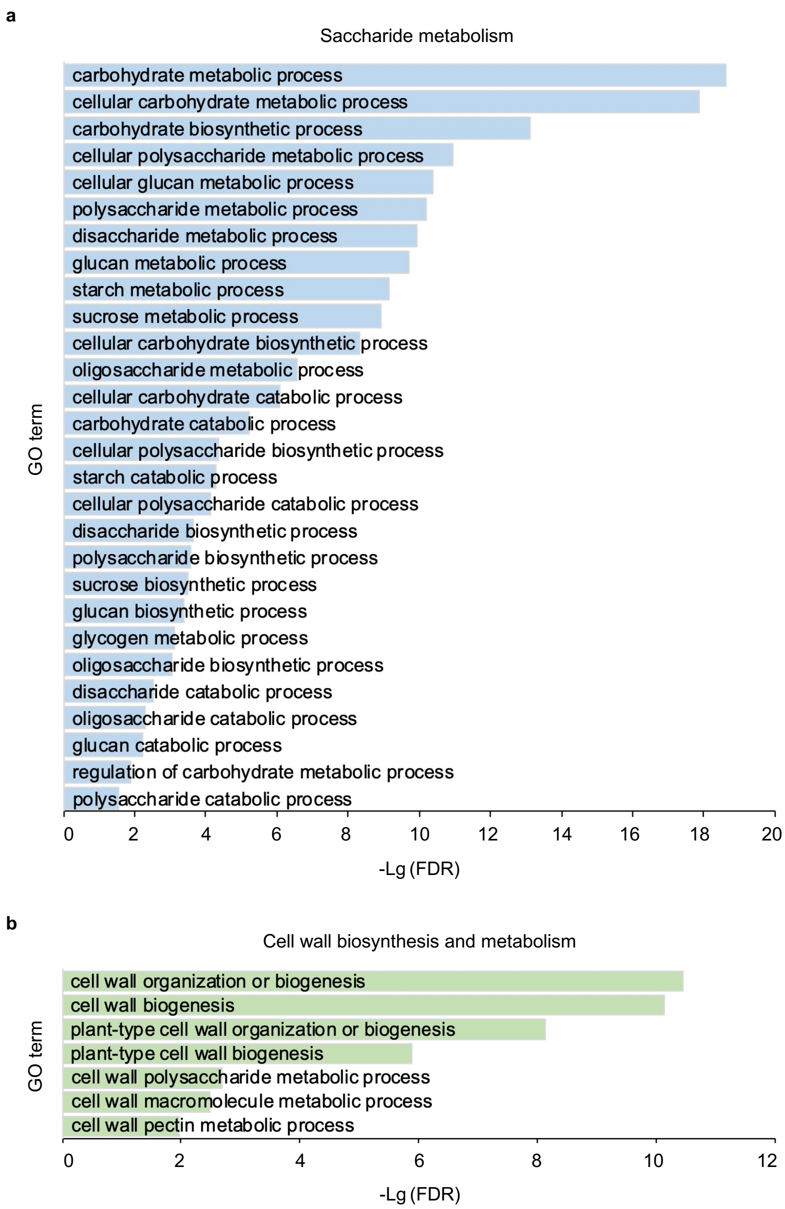

Supplemental Figure 4. Enriched GO terms related to saccharide metabolism and cell wall. **a** Enriched GO terms related to saccharide metabolism. **b** Enriched GO terms related to cell wall biosynthesis and metabolism. FDR was used to indicate significance of enrichment.

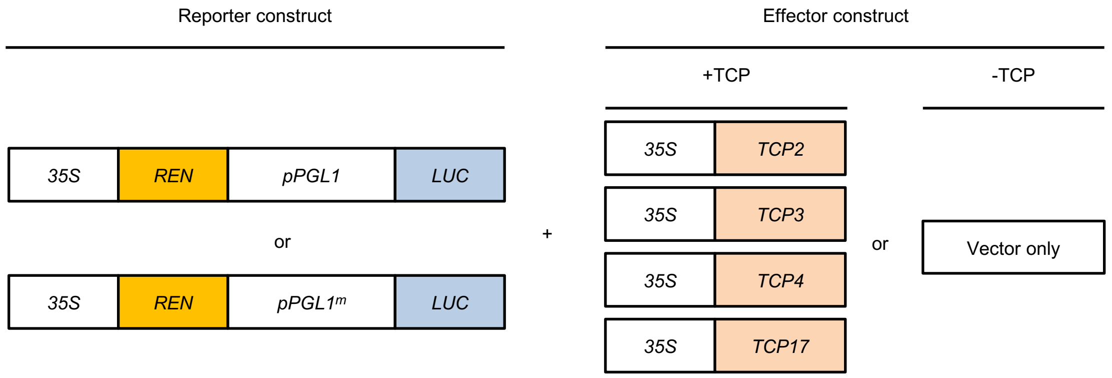

Supplementary Fig. 5 Design of the REN/LUC assay. In the reporter constructs, the native *PGL1* promoter (*pPGL1*) or a mutant version of the *PGL1* promoter (*pPGL1^m*) with the three TCP binding sites mutated and the *35S* promoter were used to drive expression of *LUC* and *REN*, respectively. The reporter and the *35S:TCP2/3/4/17* effector constructs were combined (+TCP) and used to co-infiltrate tobacco leaves. Co-infiltration of the empty vector and the reporter was used as the -TCP control.

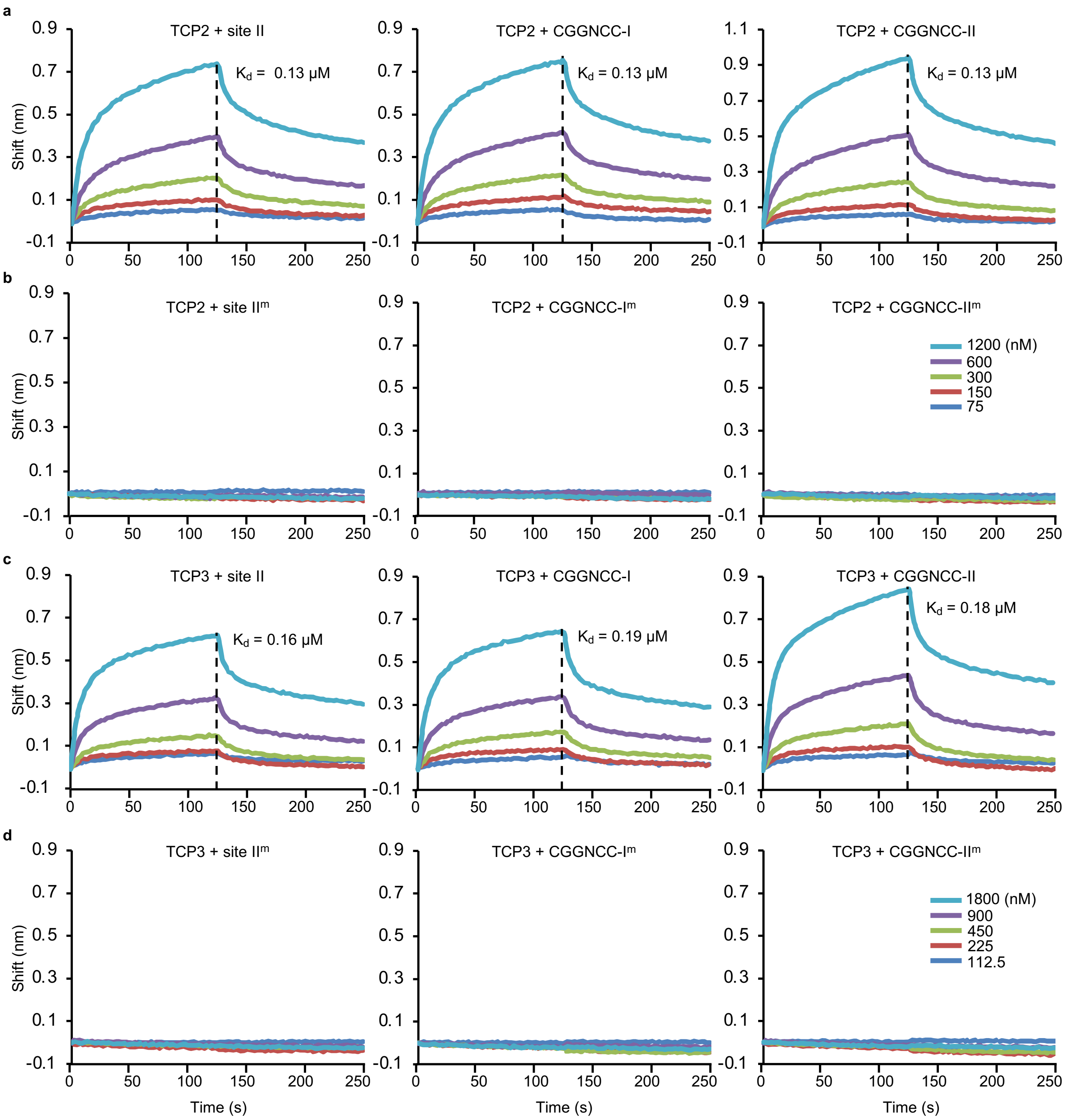

Supplementary Fig. 6 Confirmation of TCP2 and TCP3 binding to the *PGL1* promoter using BLI. The BLI assay was used to monitor the TCP2 (**a-b**) and TCP3 (**c-d**) binding kinetics to the three DNA fragments containing the CIN-TCP binding motifs (site II, CGGNCC-I and CGGNCC-II) (**a, c**) or the mutant versions (site II^{m}, CGGNCC-I^{m} and CGGNCC-II^{m} (**b**, **d**). For each combination, the concentration of TCP2 or TCP3 was varied according to the colored scheme and the DNA concentration was kept constant. The processed data curves for the association and dissociation steps are shown, divided by the vertical dashed lines. The calculated K_D values for each interaction are shown next to the curves. Source data are provided as a Source Data file.

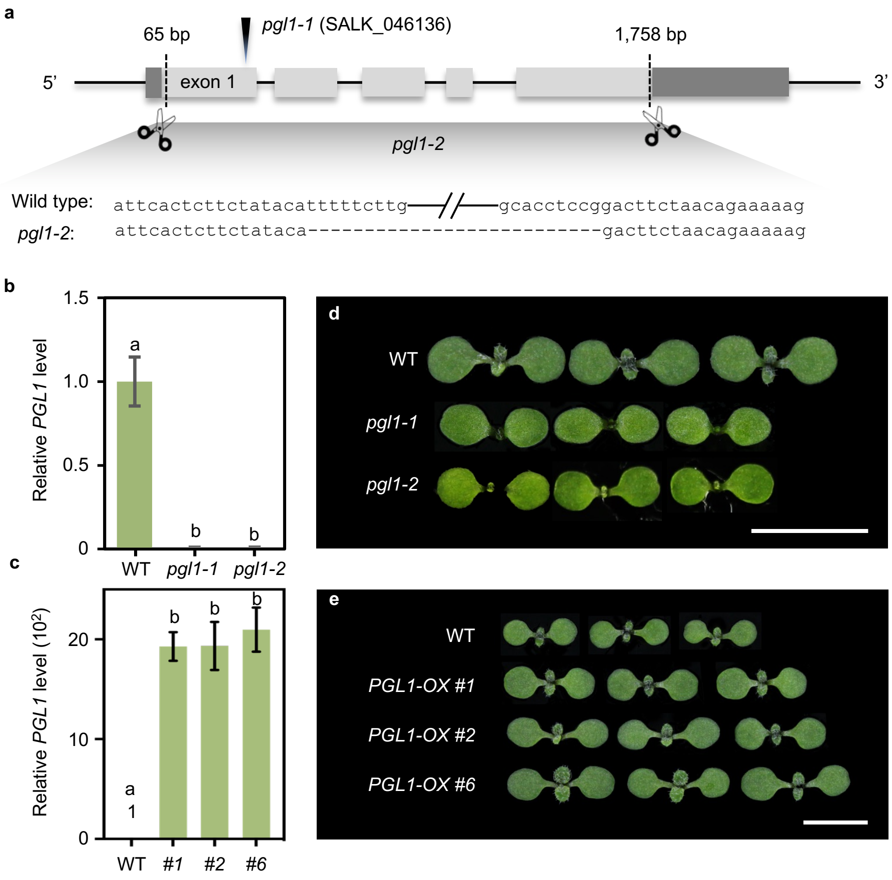

Supplementary Fig. 7 Characterization of *pgl1* and *PGL1-OX* lines. a Schematic drawing illustrating the *PGL1* gene structure and the *pgl1* mutants. Exons and UTRs are shown as light grey and dark grey horizontal boxes. The T-DNA insertion site in *pgl1-1* is indicated by the black arrow. Also shown is the scheme for generating the *pgl1-2* deletion alleles using the CRISPR/Cas9 system. The paired sgRNAs are designed to delete a 1,693 bp region in *PGL1*. Sequence comparison for a typical deletion allele with reference to the wild type allele is shown on the bottom. **b, c** Relative transcript abundance of *PGL1* in 7-day-old *pgl1* (**b**) and *PGL1-OX* (**c**) cotyledons in comparison to the wild type. The expression level was determined by RT-qPCR analysis using *ACTIN7* as the internal control and normalized to the wild type. Values are mean ± SD (n = 3 biological replicates). #1, 2, and 6 indicate independent lines. Statistical analysis was performed using one-way ANOVA with Tukey's multiple comparison test and different letters above the bars indicate statistical significance at $p < 0.001$. Source data are provided as a Source Data file. **d, e** Morphology of 7-day-old *pgl1* (**d**) and *PGL1-OX* (**e**) seedlings in comparison to the wild type. Scale bars, 0.5 cm.

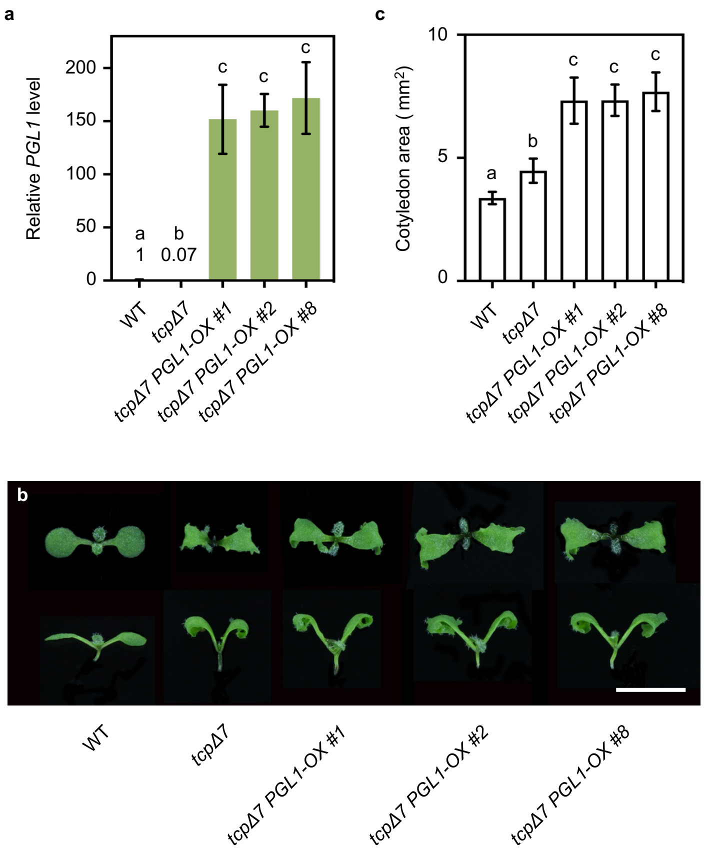

Supplementary Fig. 8 Characterization of *tcpΔ7 PGL1-OX* lines. a Relative *PGL1* transcript abundance in the cotyledons of independent *tcpΔ7 PGL1-OX* lines #1, #2, and #8. The expression level was determined by RT-qPCR analysis using *ACTIN7* as the internal control. Values are mean ± SD (n = 3 biological replicates). Source data are provided as a Source Data file. **b** Morphology of representative 7-day-old seedlings of the indicated genotypes. Scale bar, 0.5 cm. **c** Quantification of cotyledon size. Values are mean ± SD (n = 30 independent cotyledons). Statistical analysis was performed using one-way ANOVA with Tukey's multiple comparison test and different letters above the bars indicate statistical significance at $p < 0.001$. Source data are provided as a Source Data file.

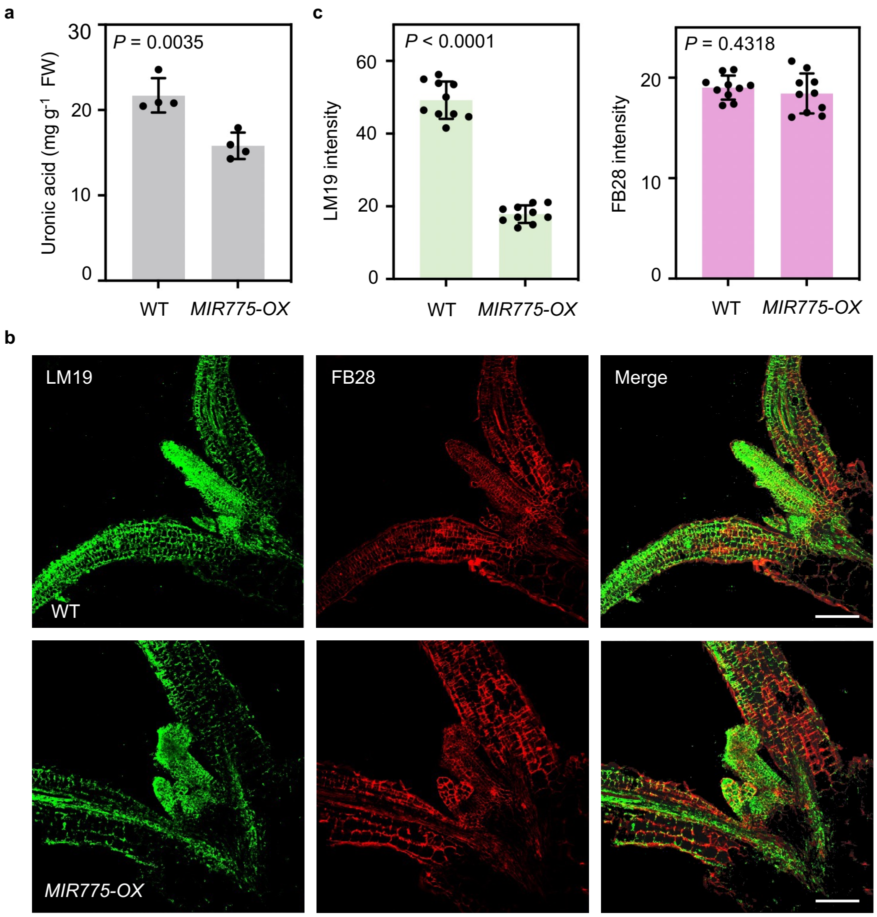

Supplementary Fig. 9 *MIR775* promotes cell expansion and represses cell wall pectin accumulation. a Chemical quantification of pectin contents in the third pair of leaves of 4-week-old plants. Values are mean ± SD (*n* = 4 independent experiments). Statistical analysis was performed using a two-tailed unpaired Student's *t* test. FW, fresh weight. Source data are provided as a Source Data file. **b** Immunolabeling analysis of sections of 7-day-old seedlings with LM19 (green) to label de-methylesterified HG and FB28 (red) to label cellulose. Scale bars, 100 µm. **c** Quantification of average intensity of LM19 and FB28. Values are mean ± SD of 10 areas (100 µm by 100 µm) from five cotyledons. ***, $p < 0.001$ by Student's *t*-test. n.s., no significance. Source data are provided as a Source Data file.

Supplementary Table 1. Oligonucleotide sequences of the primers used in this study.

For plasmid construction

Name	Sequence (5' to 3')
PGL1-OX-Fw	GGAGAGAACACGGGGGACTCTAGAATGTCATTACCTAATTCACT
PGL1-OX-Rv	TAGTAGGCCTGGTACCCTCGAGTTAGAAGTCCGGAGGTGC
TCP2-OX-Fw	GGAGAGAACACGGGGGACTCTAGAATGATTGGAGATCTAATGAA
TCP2-OX-Rv	ACTAGTAGGCCTGGTACCCTCGAGTCAGTTCTTGCCTTTA
TCP3-OX-Fw	GGAGAGAACACGGGGGACTCTAGAATGGCACCAGATAACGACCA
TCP3-OX-Rv	ACTAGTAGGCCTGGTACCCTCGAGTTAATGGCGAGAATCGGATG
TCP4-OX-Fw	GGAGAGAACACGGGGGACTCTAGAATGTCTGACGACCAATTCCA
TCP4-OX-Rv	ACTAGTAGGCCTGGTACCCTCGAGTCAATGGCGAGAAATAGAGG
TCP17-OX-Fw	GGAGAGAACACGGGGGACTCTAGAATGGGAATAAAAAAAGAAGA
TCP17-OX-Rv	ACTAGTAGGCCTGGTACCCTCGAGCTACTCGATATGGTCT
proPGL1-Fw	TCGACCTGCAGGCATGCAAGCTTACTTGGATTCAGTGTCAGATTCA
proPGL1-Rv	TATGTTTTTGGCGTCTTCCATGGTTTTTGTGAATGTCTTAGGAGATGA
PGL1-sgRNA1	AATTCACTCTTCTATACATT
PGL1-sgRNA2	CCGGACTTCTAACAGAAAAA
proPGL1-mut1-Fw	TAACAAACATCACTGAATTAAACTAAAACTTATCGATACCATCACAAGGT
proPGL1-mut1-Rv	ACCTTGTGATGGTATCGATAAGTTTTAGTTTAATTCAGTGATGTTTGTTA
proPGL1-mut2-Fw	GACTGATGGTGGAAAAGGAACTAAGTTTTGTCCGGGCACAAAGC
proPGL1-mut2-Rv	GCTTTGTGCCCGGACAAAACTTAGTTCCTTTTCCACCATCAGTC
proPGL1-mut3-Fw	GCTAGCCTCAAGATATATATCCTTACTAGATAAAACTTACCTATTTAGGCGGGTGTATCCG
proPGL1-mut3-Rv	CGGATACACCCGCCTAAATAGGTAAGTTTTATCTAGTAAGGATATATATCTTGAGGCTAGC
proPGL1-*HindIII*-Fw	CCCCTCGAGGTCGACGGTATCGATAAGCTTTCTTCAAAATTCCGTTTTTCCTCGTATG
proPGL1-*PstI*-Rv	AGTGGATCCCCCGGGCTGCAGAGGGTTTCATAGGTTTAGCCAAATCG

For RT-qPCR

Name	Sequence (5' to 3')
ACTIN7-Q-Fw	GGTGTCATGGTTGGTATGGGTC
ACTIN7-Q-Rv	CCTCTGTGAGTAGAACTGGGTGC
PGL1-Q-Fw	ATTTAAGTCCATCACCGGCTCC
PGL1-Q-Rv	CTCATACATGCAGCGTCCCA
CYCA2;3-Q-Fw	TTCACTCGTTCCCTTGCCTC
CYCA2;3-Q-Rv	AATCCATGACCGCGTCCTTT
CYCB1;2-Q-Fw	GCTCGCTTCCAATCTTCGTC
CYCB1;2-Q-Rv	TTTGCTCTCGTCGCCATGAT
CYCD3;1-Q-Fw	CAACAAATGCCACCGTCTCC
CYCD3;1-Q-Rv	CGGCAACTACTGATGGGAGG
CYCD3;3-Q-Fw	TCGTGGGTTGTGTCTGCTTC
CYCD3;3-Q-Rv	ATCTGCTGCTCTTGCACTCT
CDKB1;1-Q-Fw	GCGTGACTGGCATGTTTACC
CDKB1;1-Q-Rv	TCACCAAGACGATGACAACA
CDKB2;1-Q-Fw	CGTTCCTTCCACCACTCTCC
CDKB2;1-Q-Rv	TTGCCAGTGCTACGGAAACT
KRP1-Q-Fw	GGGACCACTAAAACACGCCA
KRP1-Q-Rv	AGGTTACGTGTGCGTGAAGT
SMR1-Q-Fw	ACCCACATCCCAAGAACACA
SMR1-Q-Rv	GGGTAACTTCTCCGCCGTTTA

For ChIP-qPCR

Name	Sequence (5' to 3')
PGL1-P1-Fw	GAAAACCGACTGATGGTGGAAAAG
PGL1-P1-Rv	GTCTTATAGGTGTGGTCGAGTCCA
PGL1-P2-Fw	CTAACACTTGTGTGTTGAGACCACT
PGL1-P2-Rv	AGTTTATTGACTGAGTTGAGCCAGG

For genotyping

Name	Sequence (5' to 3')
SALK-LBb1.3	ATTTTGCCGATTTCGGAAC
SAIL-LB3	TAGCATCTGAATTTCATAACCAATCTCGATACAC
GABI-o8760	GGGCTACACTGAATTGGTAGCTC
SM-JICSpm32	TACGAATAAGAGCGTCCATTTTAGAGTGA
wiscDSlox-P745	AACGTCCGCAATGTGTTATTAAGTTGTC
TCP2-LP	AACCCGTTTTATCAATTGTTGTG
TCP2-RP	AACCGGAATTTAACAAATCCG
TCP3-LP	ACCAAGCACGAATCATAGGTG
TCP3-RP	TTTAGGGTTTGGGATTTGGAG
TCP4-LP	GGAACGATTGCAGCGAGAGA
TCP4-RP	TCTGGGTTTTTCTTGATTGGTCA
TCP5-LP	TGAATCTGTTTTTCCTCCATCC
TCP5-RP	CTCGAAGCAGCAAAAGATGAC
TCP10-LP	AAGCAATGTCATCATCGACGG
TCP10-RP	ACGGCAAGCATTTTGAAGAGG
TCP13-LP	GATCTCCTCTGCGTTTACACG
TCP13-RP	AGTGTATTGGCTCGTGTACGG
TCP17-LP	TCTTTGGATCCTCAGATCTTCC
TCP17-RP	ATGTACCTTTGCTCGCATCAG
CCS52A2-LP	AATGGAAGGTCATCGGCTAAG
CCS52A2-RP	GGAGAAGGGAAGACATTCCAG
PGL1-LP	AATCCAACGTGCATAGCTTTG
PGL1-RP	GATTCATCAGCCTGCCTACAC
PGL1-KO-Fw	TCCTAAGACATTCACAAAAAATGTCA
PGL1-KO-Rv	TGTTCATACTTGGTTCTGAATCTGAA

For BLI

Name	Sequence (5' to 3')
SITEII-Fw	CACTGAAAGCCCATAAGGCCCGTCGATACC
SITEII- Rv	GGTATCGACGGGCCTTATGGGCTTTCAGTG
bmSITEII-Fw	CACTGAAAAAAAATAAAAAAAATCGATACC
bmSITEII- Rv	GGTATCGATTTTTTTTATTTTTTTTCAGTG
CGGNCC-I-Fw	TGGAAAAGGAACCGGGCCTTGTCCGGGCAC
CGGNCC-I-Rv	GTGCCCGGACAAGGCCCGGTTCCTTTTCCA
bmCGGNCC-I-Fw	TGGAAAAGGAACAAAAAATTGTCCGGGCAC
bmCGGNCC-I-Rv	GTGCCCGGACAATTTTTTGTTCCTTTTCCA
CGGNCC-II-Fw	CTTACTAGATAAGGCCCGCCTATTTAGGCG
CGGNCC-II-Rv	CGCCTAAATAGGCGGGCCTTATCTAGTAAG
bmCGGNCC-II-Fw	CTTACTAGATAAAAAAAACCTATTTAGGCG
bmCGGNCC-II-Rv	CGCCTAAATAGGTTTTTTTTATCTAGTAAG
