# Supplementary material for: The CIN-TCP transcription factors regulate endocycle progression and pavement cell size by promoting cell wall pectin degradation
Source: Nat Commun. 2025 May 2;16:4108. doi: 10.1038/s41467-025-59336-7 (PMC12048579; doi:10.1038/s41467-025-59336-7)
Supplement: Supplementary file 2 — Reporting Summary [file 41467_2025_59336_MOESM2_ESM.pdf]

nature portfolio

Corresponding author(s): Lei Li

Last updated by author(s): Mar 31, 2025

Reporting Summary

Nature Portfolio wishes to improve the reproducibility of the work that we publish. This form provides structure for consistency and transparency in reporting. For further information on Nature Portfolio policies, see our Editorial Policies and the Editorial Policy Checklist.

Statistics

For all statistical analyses, confirm that the following items are present in the figure legend, table legend, main text, or Methods section.

n/a	Confirmed	
☐	☒	The exact sample size (*n*) for each experimental group/condition, given as a discrete number and unit of measurement
☐	☒	A statement on whether measurements were taken from distinct samples or whether the same sample was measured repeatedly
☐	☒	The statistical test(s) used AND whether they are one- or two-sided<br>*Only common tests should be described solely by name; describe more complex techniques in the Methods section.*
☒	☐	A description of all covariates tested
☐	☒	A description of any assumptions or corrections, such as tests of normality and adjustment for multiple comparisons
☐	☒	A full description of the statistical parameters including central tendency (e.g. means) or other basic estimates (e.g. regression coefficient) AND variation (e.g. standard deviation) or associated estimates of uncertainty (e.g. confidence intervals)
☐	☒	For null hypothesis testing, the test statistic (e.g. *F*, *t*, *r*) with confidence intervals, effect sizes, degrees of freedom and *P* value noted<br>*Give P values as exact values whenever suitable.*
☒	☐	For Bayesian analysis, information on the choice of priors and Markov chain Monte Carlo settings
☒	☐	For hierarchical and complex designs, identification of the appropriate level for tests and full reporting of outcomes
☒	☐	Estimates of effect sizes (e.g. Cohen's *d*, Pearson's *r*), indicating how they were calculated

Our web collection on statistics for biologists contains articles on many of the points above.

Software and code

Policy information about availability of computer code

Data collection	The clean reads form RNA-seq analysis were mapped to the reference genome (TAIR10_Araport11) using HISAT (v2.1.0);Quantitative analysis of fluorescent intensity, luminescence were performed using ImageJ.
Data analysis	The BLI analysis uses the GatorOne software (Gator Bio). Statistical analyses were performed using Prism 8 (v8.4.0); Quantitative analysis of ploidy level was conducted using the FlowJo software (v10.4).

For manuscripts utilizing custom algorithms or software that are central to the research but not yet described in published literature, software must be made available to editors and reviewers. We strongly encourage code deposition in a community repository (e.g. GitHub). See the Nature Portfolio guidelines for submitting code & software for further information.

Data

Policy information about availability of data

All manuscripts must include a data availability statement. This statement should provide the following information, where applicable:

- Accession codes, unique identifiers, or web links for publicly available datasets
- A description of any restrictions on data availability
- For clinical datasets or third party data, please ensure that the statement adheres to our policy

RNA-seq data were deposited in Gene Expression Omnibus with the BioProject ID PRJNA971251.

Research involving human participants, their data, or biological material

Policy information about studies with human participants or human data. See also policy information about sex, gender (identity/presentation), and sexual orientation and race, ethnicity and racism.

Reporting on sex and gender	N/A
Reporting on race, ethnicity, or other socially relevant groupings	N/A
Population characteristics	N/A
Recruitment	N/A
Ethics oversight	N/A

Note that full information on the approval of the study protocol must also be provided in the manuscript.

Field-specific reporting

Please select the one below that is the best fit for your research. If you are not sure, read the appropriate sections before making your selection.

- [x] Life sciences
- [ ] Behavioural & social sciences
- [ ] Ecological, evolutionary & environmental sciences

For a reference copy of the document with all sections, see nature.com/documents/nr-reporting-summary-flat.pdf

Life sciences study design

All studies must disclose on these points even when the disclosure is negative.

Sample size	The sample size was determined to be adequate based on the statistical standard in the plant biology.
Data exclusions	No data were excluded.
Replication	All the experiments were repeated at least two times, and similar results were obtained.
Randomization	Plants were grown side-by-side randomly in the growth chamber. The seedlings were all selected for relevant experiments.
Blinding	We did not apply binding in this study because the samples are all form plants which would exhibit phenotypes under treatment.

Reporting for specific materials, systems and methods

We require information from authors about some types of materials, experimental systems and methods used in many studies. Here, indicate whether each material, system or method listed is relevant to your study. If you are not sure if a list item applies to your research, read the appropriate section before selecting a response.

Materials & experimental systems

n/a	Involved in the study	
[ ]	[x]	Antibodies
[x]	[ ]	Eukaryotic cell lines
[x]	[ ]	Palaeontology and archaeology
[x]	[ ]	Animals and other organisms
[x]	[ ]	Clinical data
[x]	[ ]	Dual use research of concern
[ ]	[x]	Plants

Methods

n/a	Involved in the study	
[x]	[ ]	ChIP-seq
[ ]	[x]	Flow cytometry
[x]	[ ]	MRI-based neuroimaging

Antibodies

Antibodies used	2F4 and LM19 monoclonal antibodies (PlantProbes, 1:500 dilution,Cat#PP-2F4, Cat#LM19; RRID: AB_2734788); Alexa Fluor 546 goat anti-rat IgG (Life Technologies, 1:800 dilution,Cat#A11081) and Alexa Fluor 546 goat anti-mouse IgG (Life Technologies, 1:800 dilution Cat#A11003); Anti-MYC Agarose Affinity Gel antibody (Sigma-Aldrich).

Validation	The antibodies used here have been commonly used for plant science related studies and validated by supplier.

Plants

Seed stocks	The Arabidopsis mutants ccs52a2 (SALK_073708) and pgl1-1 (SALK_202104) were obtained from the Arabidopsis Biological Resource Center.
Novel plant genotypes	For the PGL1-OX; tcpΔ7 PGL1-OX plants; pgl1-2.
Authentication	Verified using PCR genotyping and sequencing.

Flow Cytometry

Plots

Confirm that:

☒ The axis labels state the marker and fluorochrome used (e.g. CD4-FITC).

☒ The axis scales are clearly visible. Include numbers along axes only for bottom left plot of group (a 'group' is an analysis of identical markers).

☐ All plots are contour plots with outliers or pseudocolor plots.

☒ A numerical value for number of cells or percentage (with statistics) is provided.

Methodology

Sample preparation	Approximately 250 mg fresh leaves were cut into small pieces in ice-cold Otto I solution (100 mM citric acid, 0.5% (v/v) Tween 20), filtered through a 40 µm nylon mesh (Falcon), and centrifuged at 150g for 5 min
Instrument	the FACSVerse flow cytometer (Becton Dickinson)
Software	Quantitative analysis of ploidy level was conducted using the FlowJo software (v10.4).
Cell population abundance	Approximately 15,000 nuclei per sample were analyzed.
Gating strategy	Gating strategy is a combination of scatter parameter (FSC/SSC) with a fluorescence parameter (relative DNA intensity, PI_PE). The relative fluorescence intensity indicated DNA content and ploidy of the nuclei (2C, 4C, 8C, 16C and 32C). FACS was used to sort nuclei with different DNA content.

☒ Tick this box to confirm that a figure exemplifying the gating strategy is provided in the Supplementary Information.
